# Mandibular third molar coronectomy in older adults and its effects on oral health‐related quality of life

**DOI:** 10.1111/ger.12794

**Published:** 2024-10-27

**Authors:** Rashida N. Simons, Jerome A. Lindeboom, Jacco G. Tuk, Jan de Lange

**Affiliations:** ^1^ Department of Oral Maxillofacial Surgery Amsterdam University Medical Center Amsterdam The Netherlands; ^2^ Department of Oral Maxillofacial Surgery Ziekenhuis Amstelland Amstelveen The Netherlands

**Keywords:** coronectomy, mandibular third molar, OHRQoL, older adults

## Abstract

**Objective:**

The purpose of this study was to investigate the effect of coronectomy on postoperative quality of life in older adults.

**Background:**

Coronectomy is an alternative to complete surgical removal of a mandibular third molar that lies close to the inferior alveolar nerve.

**Materials and Methods:**

This prospective study included patients >60 years old who had an indication for coronectomy of a mandibular third molar. Patients were asked to complete the Dutch version of the Oral Health Impact Profile‐14 (OHIP‐14) daily during the first postoperative week. Postoperative pain, swelling, limited mouth opening, chewing ability and infection were also recorded. Furthermore, the effect of the impaction pattern, state of eruption, presence of preoperative pathology, patient health status according to the American Society of Anaesthesiologists score, gender, smoking on the postoperative OHIP‐14 and pain scores were investigated.

**Results:**

Thirty patients (16 males, 14 females) with a mean age of 71.2 (SD 8.3, range 60–91) years were included in the study. OHIP‐14 and pain scores were highest on the first postoperative day and gradually declined during the first postoperative week. Patients who underwent coronectomy of a fully impacted mandibular third molar had significantly higher OHIP‐14 scores on the first postoperative day than those who underwent coronectomy on a (partially) erupted mandibular third molar. We did not observe any postoperative complications up to 1 year after the surgery.

**Conclusion:**

Mandibular third molar coronectomy seems to present a valid treatment option in older adults.

## 1 INTRODUCTION

The indications for mandibular third molar removal have changed over the years, and early prophylactic removal of asymptomatic third molars is currently less common.^1^ This, combined with the fact that globally life expectancy has increased over the past two decades to 73.0 years means that means that the number of older adults with their mandibular third molars has increased.^2^, ^3^ This ultimately increases the number of patients undergoing mandibular third molar surgery at an older age.^3^

As with any oral surgery, removing the mandibular third molars affects the patient's postoperative quality of life. Third molar removal at an older age is associated with a higher risk of postoperative complications, such as alveolar osteitis, injury to the inferior alveolar nerve (IAN), trismus and intraoperative and postoperative infection.^4^, ^5^, ^6^, ^7^ Older adults are more often medically compromised and have higher American Society of Anesthesiologist (ASA) scores, which is also a risk factor for postoperative complications^3^, ^8^ that negatively affect a patient's postoperative quality of life.^9^, ^10^ Furthermore, polypharmacy is more frequent in older adults and is associated with impaired wound healing following tooth extraction.^11^

Coronectomy is an alternative procedure to complete surgical removal of the mandibular third molar. When a third molar is near the IAN, there is a risk of injuring the IAN, either by direct trauma by instruments used during surgery or compression or laceration of the IAN when the third molar roots, which lie near the IAN, are moved during surgery.^12^ Injury to the IAN leads to neurosensory disturbances of the lower lip, chin and gum area. Coronectomy significantly reduces this risk because it involves the removal of solely the crown of the tooth while leaving the roots within the alveolar bone.^13^, ^14^

The effect of coronectomy on a patient's postoperative quality of life in the younger population has been researched previously.^15^ No studies have yet assessed the impact of coronectomy on the postoperative quality of life in older adults specifically. This study aimed to determine the effect of coronectomy on the postoperative quality of life during the first postoperative week in older adults using the Oral Health Impact Profile‐14 (OHIP‐14) questionnaire, with the primary outcome measures being the mean OHIP‐14 and pain scores on each day of the first postoperative week. Secondary outcome measures included postoperative pain, swelling, trismus, postoperative bleeding, infection, neurosensory deficits and postoperative self‐care behaviours, such as using analgesics and cooling the cheek with ice on the first postoperative day. Factors potentially influencing the postoperative OHIP‐14 score (such as smoking, impaction pattern of the mandibular third molar, inclination of the mandibular third molar and state of eruption) were also evaluated. Patients were monitored for at least 1 year after the surgery, and follow‐up appointments were attended, including clinical examination and panoramic imaging.

## 2 MATERIALS AND METHODS

### 2.1 Design

This prospective study was approved by the medical ethical committee of Amsterdam University Medical Center (reference number W19_015# 19.033, Trial registration number ISRCTN15812606). Data were collected between 1 February 2019 and 31 December 2021. All patients received extensive information about the study and consented to participate. All patients agreed to complete the Dutch version of the OHIP‐14 for the first 7 days after the surgery and attend a 1‐week follow‐up appointment.^10^

### 2.2 Eligible patients

This study included older adults who were referred to the Oral and Maxillofacial Surgery Department of Amstelland Hospital for the removal of an impacted mandibular third molar. During the first visit, a panoramic radiograph was taken (Orthopantomograph® OP100 D; GE Healthcare, Dental, Tuusula, Finland). If one of the following radiographic signs associated with the third molar being near the mandibular canal were observed, computed tomography (CT; Philips Ingenuity 128 CT Scanner; Integrity Medical, Fort Myers, FL, USA) was performed to assess the precise location of the roots relative to the mandibular canal: darkening of the roots, narrowing or interruption of one or both of the white lines representing the mandibular canal, narrowing of the third molar roots, diversion of the mandibular canal.

Patients were eligible to be included in this study if the CT scan revealed that the roots were in contact with the mandibular canal, an indication for coronectomy rather than extraction.

### 2.3 Inclusion and exclusion criteria

The inclusion criteria for this study were an indication for coronectomy on a mandibular third molar, a minimum age of 60 years at the time of the surgery, and fluency in Dutch to complete the Dutch version of the OHIP‐14 questionnaire. Patients were excluded if they were <60 years old or did not have an indication for coronectomy of a mandibular third molar.

### 2.4 Surgical technique

All surgeries were performed by two oral and maxillofacial surgeons experienced in performing coronectomy. All surgeries were performed under local anaesthesia by a standardised mandibular block injection, followed by local infiltration to target the buccal nerve. The site of administration, temperature, amount of anaesthetic (articaine hydrochloride [40 mg], epinephrine [0.01 mg], 1.7‐mL syringe [Ultracain D‐S Forte; Sanofi‐Aventis Netherlands BC, Gouda, the Netherlands]) and needles (27‐gauge, 40 × 35 mm) were all standardised and followed the hospital's protocol.

First, a triangular flap was used to expose the mandibular third molar. The first incision started at the distobuccal edge of the second molar, dropped at a slightly oblique angle and then curved forward into the mandibular vestibule. The second incision started at the mandibular ramus and continued to the distobuccal part of the second molar.

Bone covering the third molar was removed using a round surgical bur until the cementoenamel junction was accessible. Subsequently, the crown of the molar was separated from the roots using a steel surgical fissure bur. The roots were then shortened to 3–4 mm below the bony margin, and mobility was checked. Thorough irrigation with sterile distilled water was performed through rotary instrumentation. Any soft tissue from the dental follicle was removed from the socket, and the wound was irrigated with a saline solution. The surgical site was primarily closed with 3/0 Undyed Vicryl Rapide (Ethicon, Somerville, MA, USA).

### 2.5 Postoperative instructions

Patients were instructed to bite down on a gauze for the first 30 minutes after surgery to stop the bleeding. If patients used antithrombotic medication (acenocoumarol, rivaroxaban, apixaban, acetylsalicylic acid and clopidogrel), the wound was checked 30 minutes after surgery to determine if the bleeding had stopped. Patients were also instructed not to rinse their mouths or spit for the first 24 hours after surgery. All patients were prescribed chlorhexidine mouthwash (0.12%, twice a day for 5 days starting the first postoperative day), postoperative antibiotics for 5 days (amoxicillin [500 mg, three times a day] or clindamycin [300 mg, three times a day] in case of an amoxicillin allergy) and analgesics (1000 mg paracetamol, four times a day). Patients received verbal postoperative instructions during the visit, written in a folder.

### 2.6 Outcome measures

The primary outcome measure was the mean OHIP‐14 and pain scores each day of the first postoperative week. The secondary outcome measures were postoperative pain, swelling, trismus, postoperative bleeding, infection, potential neurosensory deficits and postoperative self‐care behaviours (using analgesics and cooling the cheek with ice on the first postoperative day). The pattern of impaction according to the Pell and Gregory classification^16^ and inclination of the mandibular third molars was determined on the preoperative panoramic radiographs.

### 2.7 OHIP‐14 questionnaire

To assess the effect of coronectomy on postoperative quality of life, the Dutch version of the OHIP‐14 questionnaire^17^ was used. Patients were instructed to fill out the questionnaire every night around the same time and to choose the highest score they experienced that day.

The OHIP‐14 questionnaire consists of 14 questions covering seven domains about the patient's experience after surgery, which they could answer based on a 5‐point Likert scale ranging from 0 (never) to 4 (very often) (Table 1).

**TABLE 1:** Domains of the Oral Health Impact Profile‐14 questionnaire.

Domain	Item topics
Functional limitation	Trouble pronouncing words Deteriorated sense of taste
Physical pain	3 Experienced pain 4 Trouble eating and/or drinking
Psychological discomfort	5 Felt self‐conscious 6 Felt tense
Physical disability	7 Unable to eat and/or drink 8 Interrupted meals
Psychological disability	9 Trouble relaxing 10 Felt embarrassed
Social disability	11 Felt irritable 12 Experienced difficulty doing daily tasks
Handicap	13 Experienced life being less satisfactory 14 Completely unable to function

Patients were also asked to score the pain they experienced during the day on an 11‐point scale ranging from 0 to 10. Postoperative cheek swelling, limited mouth opening, chewing ability, postoperative infection and neurosensory disturbances were also recorded. Patients were also asked to record the number of analgesics they used each day of the first postoperative week.

Every patient received seven copies of the complete questionnaire, one each day of the first postoperative week. One additional question was added to the questionnaire for the first postoperative day to determine how long they kept the cheek cool with an ice pack after surgery.

### 2.8 Statistical analyses

The Shapiro–Wilk test showed that not all dependent variables were normally distributed, so non‐parametric tests were used. We used the Friedman test to assess differences between multiple measurements within one patient. The Wilcoxon signed‐rank test was used as a post‐hoc analysis. A significance level of 5% was applied, and when needed, the *P*‐value was adjusted according to the Bonferroni correction. The Kruskal–Wallis test was used to assess the effects of impaction pattern, as classified by the Pell and Gregory classification system, the inclination of the molars, the patient's health status according to the ASA score, and preoperative pathology on postoperative pain and OHIP‐14 scores. The Mann–Whitney *U*‐test was used to evaluate the association of gender, smoking and eruption status with the OHIP‐14 and pain scores. In all analyses, differences were deemed significant if *P* ≤ .05.

## 3 RESULTS

Thirty patients (16 males and 14 females) were included in this study. The mean age among the included patients was 71.2 (SD 8.3) years and ranged from 60 to 91 (Table 2). Only three participants (10%) were active smokers. Twenty‐eight patients had no symptoms related to the mandibular third molar before surgery, and two patients experienced some preoperative discomfort because of the third molar (preoperative OHIP‐14 score). Mandibular third molar characteristics are presented in Table 2. Most (47%) of the mandibular third molars were mesially inclined, 87% were not erupted, and all of the molars showed some sign of preoperative pathology.

**TABLE 2:** Demographic characteristics of the study sample.

Characteristics	*N* (%)
Sex	
Female	14 (47)
Male	16 (53)
Mean age	71.2 years (SD: 8.3)
Pell and Gregory classification	
1A	1 (3)
1B	0 (0)
1C	0 (0)
2A	0 (0)
2B	0 (0)
2C	11 (37)
3A	0 (0)
3B	1 (3)
3C	17 (57)
Inclination	
Horizontal	8 (27)
Mesial	14 (47)
Vertical	5 (16)
Distal	3 (10)
Eruption status	
(Partially) erupted	4 (13)
Not erupted	26 (87)
Smoking	
Yes	3 (10)
No	27 (90)
Preoperative pathology	
Cysts	8 (27)
Pockets	6 (20)
Caries	16 (53)
All	30 (100)

Twenty‐three patients suffered from pre‐existing medical conditions and used several medications, which are listed in Table 3.

**TABLE 3:** Medical conditions and medications used by the study sample.

	*N* (%)
Medical condition	
Cardiovascular	11 (37)
Hypertension	10 (33)
Tumour	1 (3)
Diabetes	1 (3)
Other^a^	8 (27)
No pre‐existing medical condition	7 (23)
Medication	
Anticoagulation	13 (43)
Immunosuppressive therapy	2 (7)
Antiresorptive therapy	1 (3)
Antihypertensivum	6 (20)
Other^b^	4 (13)
No medication	7 (23)

^a^ Other conditions included sleep apnoea, lung disease, inflammatory bowel disease, coronary heart disease, hypothyroidism and depression.

^b^ Other medications included proton pump inhibitors, levothyroxine.

**FIGURE 1:**
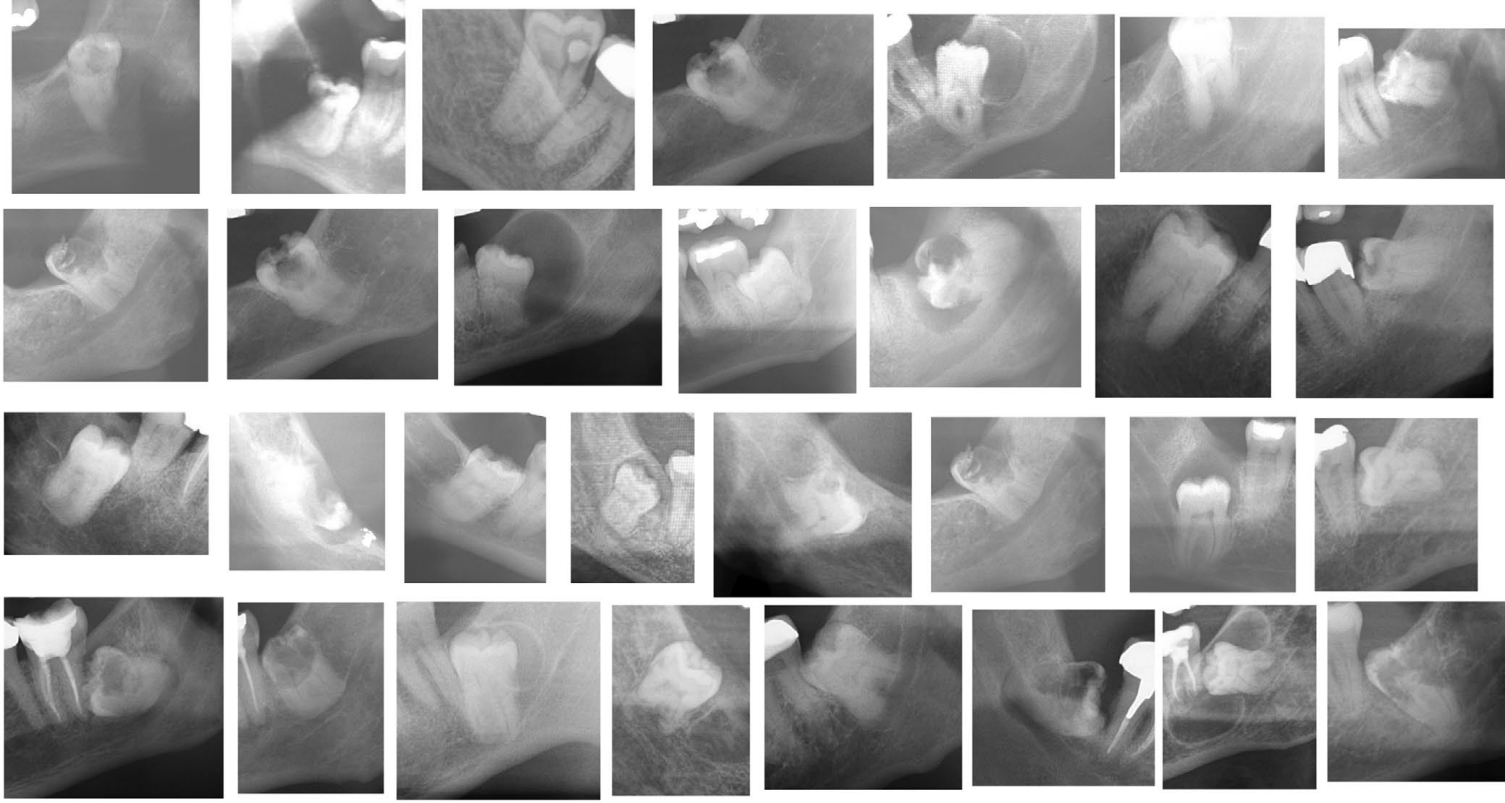
The preoperative section of the panoramic radiographs of the included patients.

Figure 1 shows the preoperative section of the panoramic radiograph of the included patients.

**FIGURE 2:**
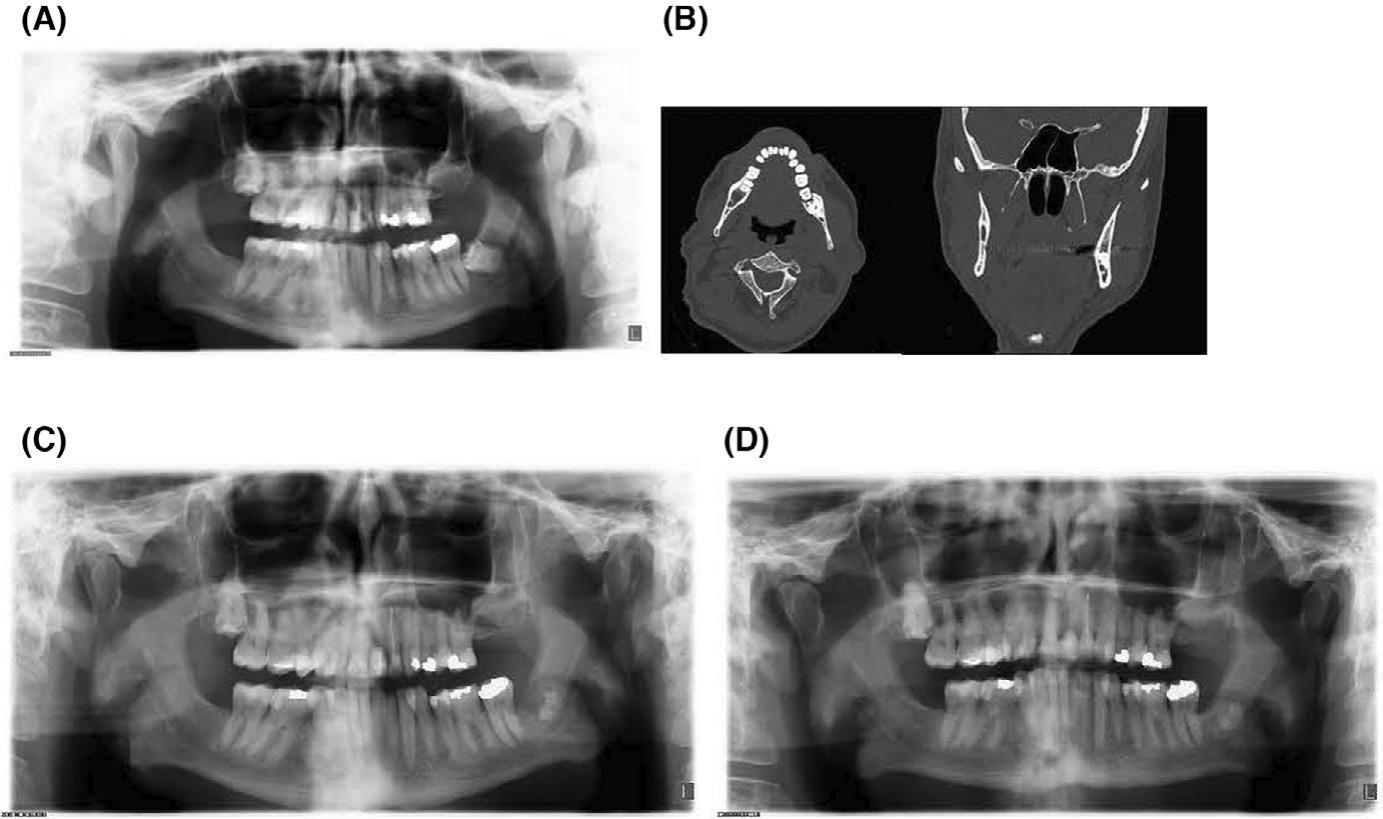
The preoperative X‐orthopantomogram (X‐OPT) (A), a preoperative CT scan (B), a postoperative X‐OPT at 6 months after coronectomy (C) and a postoperative X‐OPT at 1 year after coronectomy (D) in one of the included patients.

In Figure 2, the preoperative X‐orthopantomogram (X‐OPT) (A), preoperative CT scan (B), postoperative X‐OPT at 6 months (C) and postoperative X‐OPT at 1 year (D) are illustrated.

### 3.1 Mean OHIP‐14 and pain scores

The Friedman test indicated that the mean OHIP‐14 and pain scores from each day of the first postoperative week were significantly different (*P* < .001; Table 4). The mean OHIP‐14 and pain scores were highest on the first postoperative day and gradually declined during the first postoperative week.

**TABLE 4:** Mean Oral Health Impact Profile (OHIP) scores, pain scores and number of analgesics used after surgery on each postoperative day (brackets contain standard deviations unless otherwise indicated).

	OHIP‐14 scores	Pain scores	Number of analgesics used
POD			
1	20.2 (9.4)	6.1 (2.1)	3.8 (1.7)
2	17.4 (10.1)	4.8 (2.3)	3.9 (2.1)
3	14.9 (9.9)	4.5 (2.7)	3.7 (2.2)
4	10.9 (6.6)	3.5 (2.3)	2.9 (1.9)
5	8.7 (6.2)	3.0 (2.0)	2.9 (1.7)
6	6.6 (5.4)	2.3 (1.7)	1.9 (1.8)
7	3.2 (4.0)	1.6 (1.7)	1.3 (1.5)

Abbreviation: POD, postoperative day.

A pairwise comparison was performed using the Wilcoxon signed rank test to assess the difference between OHIP‐14 and pain scores for each day of the first postoperative week separately. The *P*‐values were adjusted for the Bonferroni correction, and differences were considered significant when *P* < .05.

All mean OHIP‐14 scores from the first seven postoperative days following coronectomy were significantly different from each other (*P* < .05). Similar results were found for the pain scores, with all scores from the first seven postoperative days following coronectomy significantly different (*P* < .05).

### 3.2 Number of analgesics used and cooling with ice

After surgery, patients used a postoperative ice pack on the cheek for an average of 5.9 hours (range 1 to 12 hours). The mean number of analgesics used is portrayed in Table 4. Most analgesics were used on the first two postoperative days and gradually declined during the week. On postoperative days 1, 2 and 3, significantly more analgesics were used than on day 6 (*P* < .05). On postoperative days 1 through 5, considerably more analgesics were used than on day 7 (*P* < .05).

### 3.3 Associations with OHIP‐14 and pain scores

The Kruskal–Wallis test was used to determine whether (i) impaction pattern according to the Pell and Gregory classification and inclination of the mandibular third molars, (ii) presence of preoperative pathology, such as cysts, a pocket distal to the adjacent second molar, caries, or preoperative complaints, and (iii) patients' health status according to the ASA score affected the mean OHIP‐14 and pain scores for each day of the first postoperative week.

There were no statistically significant differences.

The Mann–Whitney *U*‐test was used to determine whether gender, smoking and state of eruption were associated with the mean OHIP‐14 and pain scores for each postoperative day. The analysis for both gender and smoking did not reveal any statistically significant differences, but eruption status was associated with the mean OHIP‐14 score on the first postoperative day (*P* = .043). Patients who underwent coronectomy on a (partially) erupted lower third molar had a significantly lower mean OHIP‐14 score on the first postoperative day than those who underwent coronectomy on a completely impacted mandibular third molar.

### 3.4 Self‐perceived discomfort and self‐care behaviour

The majority of patients experienced limited mouth opening (80%), reduced chewing ability (100%), swelling of the cheek (96.7%) and pain (90%) on the first postoperative day. Twenty‐eight (93.3%) of the patients reported that they used the prescribed pain medication, and 11 (36.7%) patients reported that they used additional medication on the first postoperative day. On the second postoperative day, we found an increase in patients who experienced limited mouth opening (86.7%) and those who used additional medication (43.3%). From the third through the seventh postoperative day, the number of patients who used prescribed or additional medication or experienced limited mouth opening, reduced chewing ability, swelling of the cheek and pain decreased (Table 5). On the seventh postoperative day, eight (26.7%) of the patients reported that they no longer experienced any problems due to the surgery.

**TABLE 5:** Number of patients reporting self‐care behaviours (brackets contain percentage of the sample).

	POD 1	POD 2	POD 3	POD 4	POD 5	POD 6	POD 7
Questions							
Did you use the prescribed pain medication?	28 (93)	27 (90)	25 (83)	25 (83)	20 (67)	17 (57)	12 (40)
Did you use additional medication?	11 (37)	13 (43)	9 (30)	9 (30)	12 (40)	6 (20)	6 (20)
Did you experience limited mouth opening?	24 (80)	26 (87)	25 (83)	20 (67)	19 (63)	17 (57)	15 (50)
Did you experience reduced chewing ability?	30 (100)	28 (93)	22 (73)	22 (73)	22 (73)	18 (60)	12 (40)
Did you experience a swollen cheek?	29 (97)	29 (97)	27 (90)	25 (83)	25 (83)	19 (63)	12 (40)
Did you experience pain as a result of the surgery?	27 (90)	25 (83)	22 (73)	18 (60)	16 (53)	14 (47)	12 (40)
Did you experience any problems at all?	0 (0)	0 (0)	0 (0)	2 (7)	1 (3)	4 (13)	8 (27)

Abbreviation: POD, postoperative day.

### 3.5 Postoperative complications

No postoperative infections, bleeding, alveolitis or neurosensory disturbances were observed during the first postoperative week up to 1 year after the surgery.

## 4 DISCUSSION

This study investigated older adults' postoperative quality of life in the first week following coronectomy. We found that OHIP‐14 and pain scores were highest on the first postoperative day and gradually declined during the first postoperative week, reflecting steady improvement in OHRQoL. Factors such as impaction pattern, inclination, smoking and gender were not associated with the postoperative OHIP‐14 and pain scores, but the state of eruption did; patients with completely impacted mandibular third molars had a significantly higher mean OHIP‐14 score on the first postoperative day than those without impacted mandibular third molars.

This is the first study to investigate the effect of coronectomy in older adults. It has some limitations. First, its design did not include a control group consisting of patients in a younger age group. Because of this, we cannot attribute our findings to the age of the patients alone. Additionally, because older adults with indications for coronectomy are rare, the sample size was relatively small, limiting the findings' precision and generalisability. Of course, the lack of prior research on this subject complicates the comparison of our findings with existing literature.

However, a few studies have investigated the effect of coronectomy on the postoperative quality of life of patients in a younger age group. Manor et al.^18^ studied the impact of coronectomy on postoperative quality of life in 24 patients (mean age 28). Similar to our study, quality of life scores were highest on the first day and declined over the first postoperative week.^18^ Tuk et al.^15^ also assessed the impact of coronectomy on the postoperative quality of life in 50 patients (mean age 25 years) using the OHIP‐14 questionnaire. They found mean OHIP‐14 (26.4 SD 8.7) and pain scores (6.4 SD 2.1) to be highest on the first day and decreasing over the week. Tuk et al.^15^ reported higher OHIP‐14 and pain scores for each day of the first postoperative week than we did. We also compared our findings to data from a previous crossover study that assessed the difference in the postoperative quality of life following coronectomy and complete surgical removal. The included patients were younger, with a mean age of 24.6 years (18–42 years). Again, we found lower OHIP‐14 and pain scores each day of the first postoperative week following coronectomy than in that study.

In previous studies, higher age was associated with the prevalence of postoperative complications and persisting postoperative pain.^3^, ^15^ Existing theories suggest that delayed inflammatory response and impaired angiogenesis can negatively impact the healing process in older adults.^19^ Despite this, we found lower OHIP‐14 and pain scores than previous studies assessing patients in a younger age group. Tuk et al.^15^ also failed to observe gender and impaction pattern differences in postoperative quality of life.

Older age is a known risk factor for postoperative complications after the complete removal of mandibular third molars.^3^, ^4^, ^20^ Baensch et al.^3^ found a significantly higher incidence of postoperative bleeding after third molar removal in patients over the age of 65 years than in younger patients. They also found that older adults had to undergo a second surgery more frequently due to complications such as postoperative bleeding, infection or the retention of a root of the mandibular third molar.^3^ Pogrel et al.^20^ found that the incidence of postoperative complications (such as infection, jaw fractures and possible periodontal complications related to the adjacent second molar) increased with age and that the recovery process was slower in older patients. Van Wijk et al.^10^ assessed the postoperative quality of life following the complete surgical removal of mandibular third molars. They found that the presence of postoperative complications negatively affected postoperative oral health‐related quality of life.

Many mandibular third molars that need to be extracted at an older age present with preoperative pathologies, and these increase the risk of postoperative complications.^4^, ^21^ A higher ASA classification health status score is associated with higher intraoperative and postoperative complications risk.^3^ Considering that the included patients in this study all had some form of preoperative pathology and 76.7% of them had a medical condition, it is remarkable that we found no intraoperative or postoperative complications up to 1 year after the surgery. One factor that could contribute to these findings is that all patients were prescribed antibiotics for the first five postoperative days. A recent systematic review showed that patients who receive antibiotic therapy after coronectomy have lower postoperative infection rates, but no consensus has been reached on this particular subject.^22^, ^23^

Most patients in the current study had a medical condition or were on medication contraindicating non‐steroidal anti‐inflammatory drugs (NSAIDs) as analgesics after surgery. For this reason, all patients were prescribed paracetamol (acetaminophen) after the surgery. In assessing the efficacy of multiple oral analgesics after third molar surgery, Derry et al.^24^ found that paracetamol is less effective than ibuprofen or a combination of two analgesic drugs, such as ibuprofen and paracetamol. Still, it remains a suitable option for mild‐to‐moderate postoperative pain following third molar surgery.^25^ Because paracetamol has few adverse drug interactions and has very few contraindications for its use, it is an excellent alternative analgesic for medically impaired patients with contraindications for the use of NSAIDs.

In conclusion, mandibular third molar coronectomy seems to present a valid treatment option in older adults. Pain and OHIP‐14 scores were highest on the first postoperative day, and the patient's condition slowly improved during the first postoperative week. No significant postoperative complications were observed up to 1 year after the surgery. Further research should include larger study samples and control groups to confirm these findings and further explore the benefits of coronectomy in older populations.

## AUTHOR CONTRIBUTIONS

All authors contributed to the study design, data collection, analysis and writing.

## CONFLICT OF INTEREST STATEMENT

The authors have no conflicts of interest to declare.

## ETHICS STATEMENT

The study received ethical approval from the medical ethical committee of Amsterdam University Medical Center (reference number W19_015# 19.033).
